# Assembly of Hepatocyte Spheroids Using Magnetic 3D Cell Culture for CYP450 Inhibition/Induction

**DOI:** 10.3390/ijms18051085

**Published:** 2017-05-18

**Authors:** Pujan K. Desai, Hubert Tseng, Glauco R. Souza

**Affiliations:** 1 Nano3D Biosciences, Houston, TX 77030, USA; pujandesai@gmail.com (P.K.D.); htseng@n3dbio.com (H.T.); 2 Department of Internal Medicine, University of Texas Health Science Center at Houston, Houston, TX 77030, USA

**Keywords:** hepatocyte, liver, metabolomics, in vitro methods, high-throughput

## Abstract

There is a significant need for in vitro methods to study drug-induced liver injury that are rapid, reproducible, and scalable for existing high-throughput systems. However, traditional monolayer and suspension cultures of hepatocytes are difficult to handle and risk the loss of phenotype. Generally, three-dimensional (3D) cell culture platforms help recapitulate native liver tissue phenotype, but suffer from technical limitations for high-throughput screening, including scalability, speed, and handling. Here, we developed a novel assay for cytochrome P450 (CYP450) induction/inhibition using magnetic 3D cell culture that overcomes the limitations of other platforms by aggregating magnetized cells with magnetic forces. With this platform, spheroids can be rapidly assembled and easily handled, while replicating native liver function. We assembled spheroids of primary human hepatocytes in a 384-well format and maintained this culture over five days, including a 72 h induction period with known CYP450 inducers/inhibitors. CYP450 activity and viability in the spheroids were assessed and compared in parallel with monolayers. CYP450 activity was induced/inhibited in spheroids as expected, separate from any toxic response. Spheroids showed a significantly higher baseline level of CYP450 activity and induction over monolayers. Positive staining in spheroids for albumin and multidrug resistance-associated protein (MRP2) indicates the preservation of hepatocyte function within spheroids. The study presents a proof-of-concept for the use of magnetic 3D cell culture for the assembly and handling of novel hepatic tissue models.

## 1. Introduction

Drug-induced liver injury is a major concern in drug discovery, as one of the major causes of market withdrawals and attrition [1,2,3]. The fact that a large percentage of hepatotoxic liabilities are found in clinical trials and post-approval suggests the inadequacy of preclinical screening. Discovering such liabilities could reduce adverse toxic events and costs related to the progress of a compound bound to fail.

The major limitation in improving accuracy in preclinical screening is the lack of predictive models of drug-induced liver injury (DILI). Animal models, while generally representative of human physiology, still fail to predict about half of hepatoxicities seen in humans [4], likely due to differences in biology and therapeutic efficacies, and a poor reflection of genetic diversity typically seen in the human patient population [5,6,7]. In general, animal models also suffer from significant costs, low throughput, and ethical concerns [8,9,10], which can influence decisions on compound progression or attrition despite potential liabilities.

In vitro models have lower costs and higher throughput, but improvements in their predictive accuracy are still needed. Conventional hepatocyte monolayers are often used but these cells can de-differentiate and lose phenotype and function over long-term culture (>72 h) [11]. This is likely due to a two-dimensional (2D) environment that weakly represents the native tissue environment, as monolayers are seeded on rigid substrates with limited cell–cell and cell–extracellular matrix (ECM) interactions, and a unidirectional exposure to compounds [12,13,14,15]. Hepatocyte suspensions, while avoiding rigid substrates, suffer from an even faster deterioration in function and viability, as well as handling issues [16].

As a result, this study explores three-dimensional (3D) hepatocyte cultures for cytochrome P450 (CYP450, CYP) induction/inhibition that could potentially replicate native tissue environment and function similarly to in vivo tissue [17], while having similarly high throughput and low costs as monolayer cultures. There are several approaches that have been taken to measure CYP in a 3D in vitro setting that represents the liver microenvironment. “Lab-on-a-chip” approaches have been used to mimic the microfluidics and spatial organization of the liver, but achieving such a perfectly representative environment is a long and low-throughput process [18,19,20,21,22]. On the other end of the spectrum, multicellular spheroids, wherein cells aggregate and self-assemble into in vivo-like tissues, may be far simpler, but are widely used for their scalability, high throughput, the lack of xenotypic or synthetic substrates, and the ability to assay them for CYP activity [23,24,25]. Spheroids in general can be formed using a number of techniques, including bioreactors (rockers, stirrers, spinners, etc.) [26,27,28], hanging drop plates [29], micropatterned plates [30], and non-binding round bottom plates [31]. An appropriate spheroid culture system for DILI screening must be able to efficiently produce large numbers of spheroids with reproducibility, fit into existing high-throughput workflows, and be physiologically similar to the native liver.

**Figure 1:**
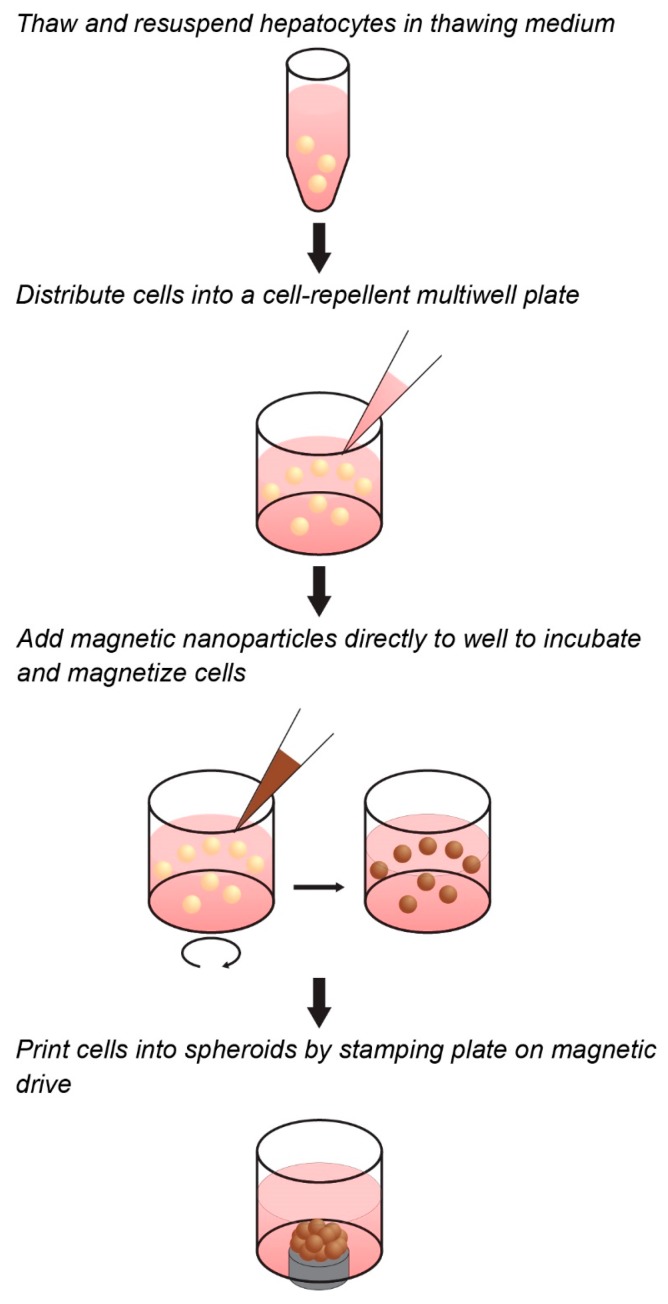
Workflow of magnetic three-dimensional (3D) cell culture. Cells can be magnetized in suspension in cell-repellent multiwell plates prior to spheroid assembly. The cells are mixed with the magnetic nanoparticles and incubated for a short time to allow adequate and uniform attachment. Magnets under the well aggregate the cells into spheroids.

Towards the goal of generating a predictive spheroid assay for CYP activity with high throughput, this study presents a model based on magnetic 3D cell culture, in which cells are magnetized, then aggregated into spheroids using mild magnetic forces (Figure 1) [32]. Magnetization is accomplished by the electrostatic attachment of a magnetic nanoparticle assembly—consisting of gold, iron oxide, and poly-l-lysine—to the cell membrane. Magnetized cells are then distributed into cell-repellent plates, where they are aggregated into spheroids by placing plates atop an array of magnets that rapidly attract magnetized cells to the bottom of the well to form a spheroid. Once aggregated, these cells organize to build a larger 3D environment that replicates native tissue. In using magnetic forces to aggregate cells, spheroid formation is rapid, easy, and does not need specialized equipment or media, while the magnetization of spheroids allows for them to be held down with magnetic forces during routine processing without attachment to a stiff substrate that could affect cell behavior. Moreover, as magnetization works at the individual cell level, and spheroid size is determined only by cell number and a fixed magnetic field shape, small spheroids can be reproducibly printed to take advantage of scarce cell sources. These attributes make this platform ideal for the high-throughput screening of CYP induction/inhibition in hepatocyte spheroids, as has been demonstrated for many other tissues [28,32,33,34,35,36,37,38,39,40,41,42,43,44,45,46,47,48].

In this study, we validate a high-throughput spheroid assay for CYP activity using magnetic 3D cell culture. This study uses primary human hepatocytes, which are typically cultured in monolayers and suspensions. We first used immunohistochemistry to characterize the spheroids for albumin, a marker of liver function used extensively in the clinic, and multidrug resistance-associated protein (MRP2), an apical membrane protein responsible for the Phase III transport of conjugated xenobiotics into the bile canaliculi [49]. After first characterizing hepatocyte spheroids, we looked at CYP induction/inhibition and viability in response to known inducers and inhibitors. The resulting assay will serve as the foundation for in vitro CYP assays in spheroids that better replicate native liver structure and function.

## 2. Results

### 2.1. Spheroid Characterization

**Figure 2:**
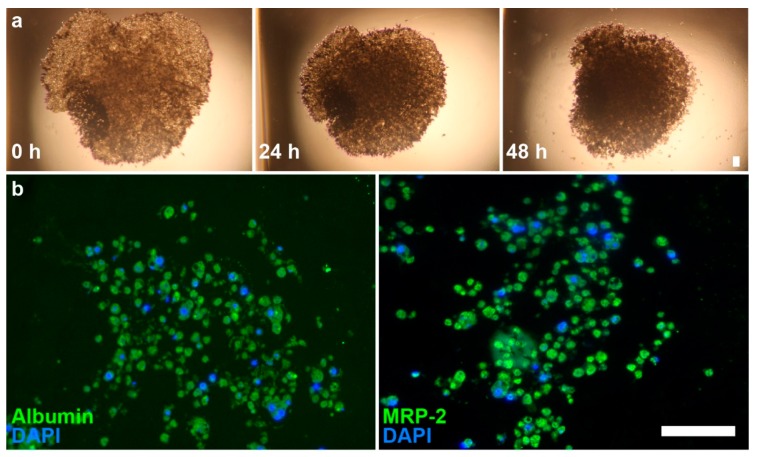
Brightfield images of primary hepatocyte spheroids and immunofluorescence images of spheroid sections. (**a**) Images of hepatocyte spheroids at 0, 24, and 48 h show contraction of structure; (**b**) Immunofluorescence shows presence of albumin (left, green) and MRP2 (right, green) on sections. Nuclei (blue) were counterstained with DAPI. Scale bar = 50 µm.

Hepatocytes quickly aggregated into spheroids at Day 0, after 1 h on the magnet drive. Over 48 h, there is a clear contraction of the structures, wherein the spheroids initially aggregate into a disc-shaped structure in the shape of the magnetic field and gradually contract into a spheroid due to cell–cell interactions (Figure 2). Immunohistochemical staining showed a loose, highly cellularized structure that stained positively for albumin and MRP2.

### 2.2. CYP Activity

**Figure 3:**
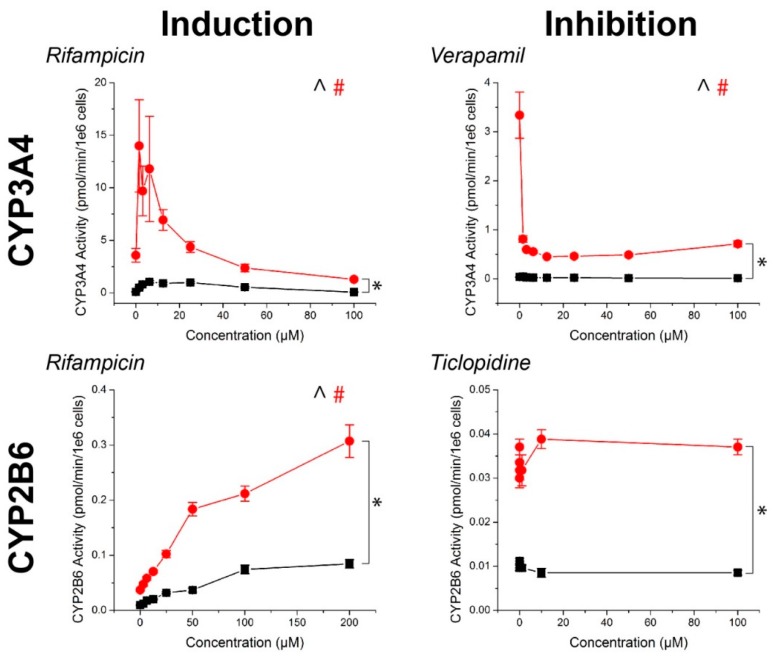

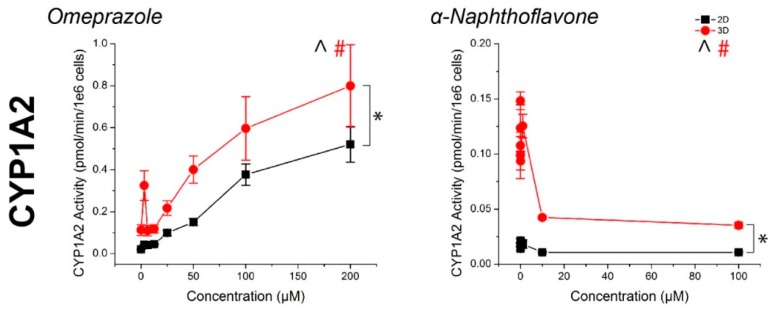
CYP450 activity in primary human hepatocytes in response to known inducers and inhibitors of CYP3A4, CYP2B6, and CYP1A2. Aside from ticlopidine, CYP activities were significantly induced and inhibited as expected. In all cases, higher CYP450 activity was observed in 3D than in 2D. ^, #: *p* < 0.05 effect of concentration on activity. *: *p* < 0.05 difference in activity between 2D and 3D. Error bars represent standard error.

**Figure 4:**
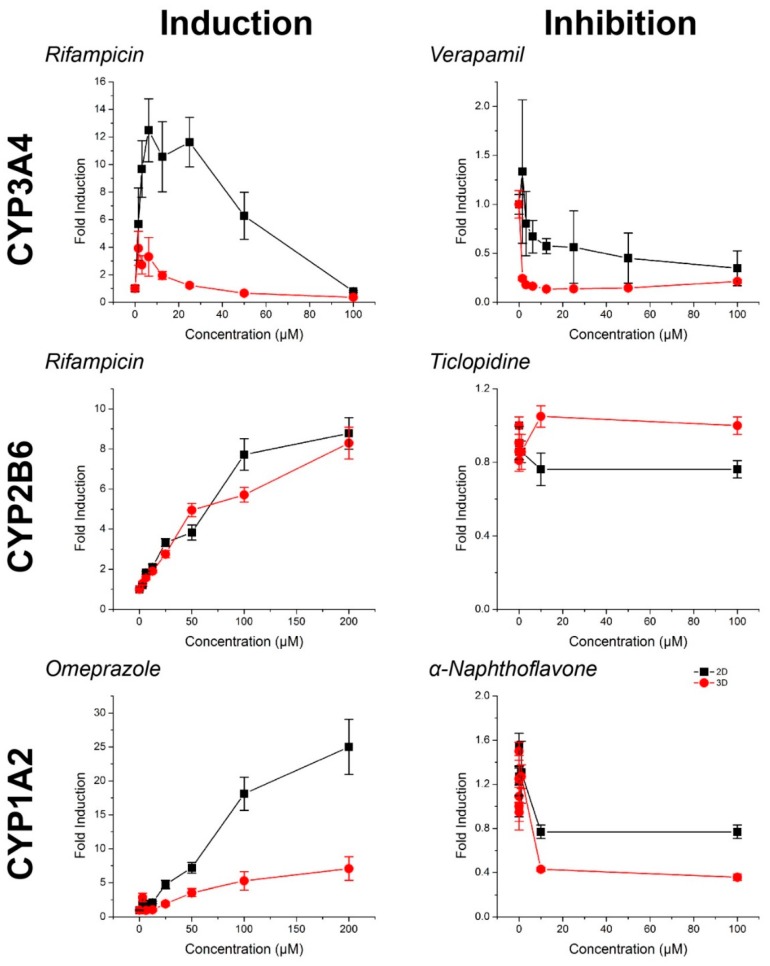
CYP450 fold induction and inhibition in primary human hepatocytes in response to known inducers and inhibitors of CYP3A4, CYP2B6, and CYP1A2, normalized to control. Higher or comparable CYP450 fold induction was observed in 2D compared to 3D. Aside from ticlopidine, where there was no significant inhibition, greater CYP450 fold inhibition was observed in 3D than in 2D. Error bars represent standard error.

For all compounds, hepatocyte spheroids showed a significantly higher baseline activity with no drugs added over monolayers (Figure 3). CYP activity was significantly induced or inhibited in a manner consistent with expectations for all drugs except ticlopidine in both monolayers and spheroids (See Table S1 for *p*-values). When this same data was converted to fold changes in CYP activity, in response to rifampicin, monolayers showed a greater fold induction in CYP3A4 activity, but similar levels of induction in CYP2B6 activity than spheroids (Figure 4). CYP1A2 induction was also higher in monolayers compared to spheroids in response to omeprazole. For the CYP inhibitors verapamil and α-napthoflavone, there was a greater fold inhibition in spheroids compared to monolayers.

### 2.3. Spheroid Viability

**Figure 5:**
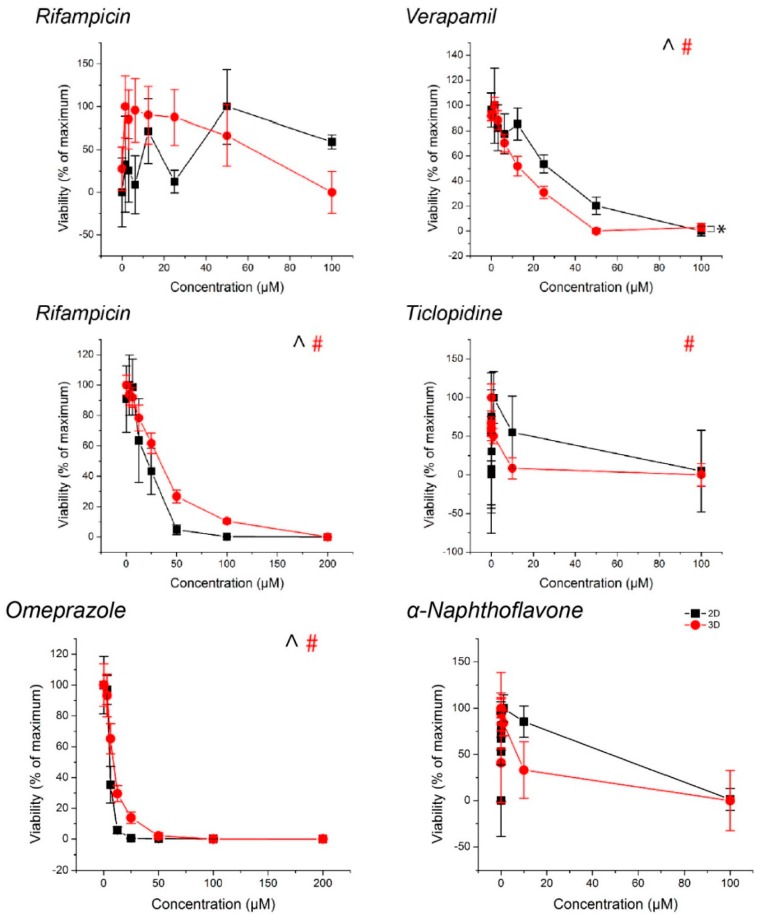
Viability of hepatocyte spheroids and monolayers, normalized to control. ^, #: *p* < 0.05 effect of concentration on viability. *: *p* < 0.05 difference in viability between 2D and 3D. Error bars represent standard error.

With the exception of rifampicin in the CYP3A4 replicates and α-napthoflavone in the CYP1A2 replicates in spheroids, monolayers exposed to ticlopidine, cytotoxic responses were observed with all drugs (Figure 5, see Table S2 for *p*-values). With the inducers rifampicin in CYP2B6 replicates and omeprazole, there was no observed decrease in CYP activity despite toxicity.

## 3. Discussion

The goal of this study was to demonstrate the ability to assay CYP activity in spheroids. We successfully printed spheroids using hepatocytes that remained intact, viable, and functional after five days of culture, as demonstrated by both CYP activity and the presence of albumin and MRP2 in the spheroid. After three days of exposure to compounds, spheroids had higher baseline CYP activity than monolayers and responded to known CYP inducers and inhibitors as expected. The result of this study is a spheroid assay for CYP induction/inhibition with a higher baseline activity and more representative environment than monolayers that can serve as the foundation for high-throughput screening of hepatotoxic liabilities.

We showed competent spheroids that formed as expected. Hepatocyte spheroids removed from the magnetic field contracted over the course of 48 h in culture. Spheroid contraction has been seen in a previous study of magnetically 3D bioprinted spheroids [43], which showed that contraction in absence of the magnetic field reflected cell viability and cell–cell interaction within the spheroid. Positive staining for albumin and MRP2 indicated the maintenance of hepatocyte function within the spheroids (Figure 2). Spheroid size could be further reduced with smaller cell numbers to make use of scarce cell sources (i.e., primary human hepatocytes) and limit any potential hypoxic effects. Overall, these results demonstrated our success in forming competent hepatocyte spheroids.

An important difference between this study and previous studies with magnetic 3D cell culture was the method of magnetization. Rather than use the typical method of magnetizing adherent cells in flasks with an overnight static incubation [28,32,33,34,35,36,37,38,39,40,41,42,43,44,45,46,47,48], we developed a new protocol that magnetizes unadhered cells in suspension. This method is advantageous over the previous method for several reasons. From thaw, we were able to assemble hepatocyte spheroids over a shorter time period (1–2 h), with magnetic aggregation ensuring close cell–cell contact. Given the quick deterioration of hepatocyte phenotype in suspension or with attachment to a stiff substrate [16], the immediate assembly of these spheroids helped to avoid these worries. Additionally, cryopreserved primary hepatocytes typically exhibit very poor adherence, even with collagen coating, and separation of non-adherent cells. Given the cost and scarcity of primary hepatocytes, this method is valuable in utilizing non-adherent cells which may otherwise be viable. The success of this new magnetization protocol was demonstrated by the lack of cells outside of the spheroid (Figure 2), indicating an efficient magnetization and aggregation of cells.

Hepatocyte spheroids showed significantly higher baseline CYP activities in spheroids than monolayers, which is consistent with previous comparisons in CYP activity [50], it should be noted that both CYP1A2 and CYP2B6 baseline activities were low when looking at raw pmol/min/10^6^ cells values (Figure 3). While the magnitudes of induction of these enzymes measured from the same spheroids were not seemingly affected (Figure 4), the lack of inhibition may have been due to an already low basal value, particularly with regards to the effects of ticlopidine, where no significant effect was found on CYP2B6 activity. Combined with the high baseline value of CYP3A4 activity, and its inhibition with verapamil, the low activities of CYP1A2 and CYP2B6 activity suggest that this batch of cells had intrinsically low activity. These results reflect a limitation of primary hepatocytes due to their variability in source. Cell lines, such as HepaRG [51], and induced pluripotent stem-cell derived (iPS) hepatocytes, may offer more consistent results, but issues in the representation of function and phenotype persist.

The influence of cell source on CYP activity can also be seen by the fact that CYP3A4 activity was reduced at high rifampicin concentrations, falling below the basal activity of the controls at 100 μM (Figure 3). No significant cytotoxic effect was found, and there was no such effect for other CYP enzymes where a significant cytotoxic effect was present. This was inconsistent with previous experiments using iPS-hepatocytes where there was a similar increase in baseline activity between spheroids and monolayers, but a monotonic induction of CYP3A4 activity with increasingly higher concentrations of rifampicin (Figure S1). These results demonstrate that cell source is an important factor to consider in screening compounds for CYP inhibition/induction. Regardless, hepatocyte spheroids were found to have higher baseline CYP activity than hepatocyte monolayers.

The duration of culture and compound exposure may have also influenced CYP activity. CYP activity was assayed at Day 5, after two days of culture and three days of compound exposure, a protocol which has been previously used in literature [52]. Different lengths of culture time and compound exposure could affect levels of CYP activity as shown in literature [24,53]. Moreover, assaying at a single point in time may have missed more acute changes in CYP activity. The luminescent endpoint for CYP activity (P450-Glo, Promega, Madison, WI, USA) is non-lytic and could theoretically be used to assay CYP activity over the course of the experiment. Such an experiment could help characterize hepatocyte maturation or loss of phenotype in spheroids, as well as provide context for discrepancies between viability and CYP induction/inhibition. The ability to measure both viability and CYP activity separately was confirmed by the varied toxic responses in relation to CYP inhibition/induction. These results demonstrate that these endpoints could be tested in one assay, rather than require two separate assays on different spheroids. Other endpoints that could be tested include albumin and urea [54]. Further development and investigation of multiplexing with these spheroids should be explored in future experiments.

The resulting assay of this study forms the foundation for high-throughput screening of DILI in hepatocyte spheroids. The results of this assay are similar to other hepatocyte assays in 3D culture in that they showed expected responses with known inducers and inhibitors [22,25,55]. What separates this assay from others is the rapid assembly of spheroids for high-throughput applications, and the results of this study demonstrate the ability to assay these spheroids and multiplex different endpoints. While this study used just one cell type, the liver contains many cell types. To achieve a higher order of complexity and representation of the liver, non-parenchymal cells can be added in co-culture to improve the accuracy of these models, such as other liver-derived cells (Kupffer, stellate, endothelial, etc.) and cell lines (i.e., 3T3-J2) [30,56]. Magnetic 3D cell culture has been shown to be highly amenable to co-cultures with past models of lung [38], heart valve [41], and adipose tissue [37]. These methods could potentially be used to add more cell types into spheroids to recapitulate liver tissue. Future experiments with this hepatocyte model should consider these factors and the cost–benefit tradeoff for high-throughput screening.

## 4. Materials and Methods

### 4.1. Magnetization and Culture of Primary Hepatocytes

Cryopreserved hepatocytes (BioreclamationIVT, Baltimore, MD, USA) were thawed as per manufacturer’s instructions in thawing media (BioreclamationIVT). For these experiments, one lot of cells was used for consistency in baseline CYP activity. Once thawed, these cells were then seeded into a cell-repellent 384-well plate (CELLSTAR^®^, Greiner Bio-One, Frickenhausen, Germany) at 10,000 cells/well. To each well, 2 µL of the magnetic nanoparticles (NanoShuttle-PL, Nano3D Biosciences, Houston, TX, USA) was added to magnetize cells. Cells were incubated with the magnetic nanoparticles at 37 °C for 1 h to allow its binding to the cell membrane. After magnetization, magnetized hepatocytes were printed into spheroids by stamping the plate atop a magnetic drive of 384 magnets positioned under each well. These magnets (0.0625 in. outer diameter) were cylindrical neodymium magnets that in this setting, through a plate thickness of 1 mm, apply a magnetic field of approximately 800 G on the cells. By placing the magnet underneath the well, spheroids were formed by the magnetic field aggregating the magnetized cells at the bottom of the well. The plate was left on magnet for 48 h to produce competent spheroids, after which the plate was removed from the magnet to culture.

For monolayers, thawed hepatocytes were seeded at a concentration of 75,000 cells/well onto a 96-well plate (Greiner Bio-One) coated with collagen type I (Sigma-Aldrich, St. Louis, MO, USA). The plate was coated by adding collagen type I at a concentration of 5 µg/cm^2^ surface area to incubate for an hour at room temperature under ultraviolet light exposure. Before seeding, the collagen solution was aspirated and the plate was washed with phosphate buffered saline (PBS, pH~7.4).

At 24 h of culture for both monolayers and spheroids, thawing media was replaced with maintenance media (BioreclamationIVT), which was subsequently replaced at 48 h with a serum-free induction media (BioreclamationIVT). At the same time that induction media was added, CYP inducing (rifampin, omeprazole) and inhibiting (verapamil, ticlopidine, α-napthoflavone) drugs (Sigma-Aldrich) were added at various concentrations (*n* = 3 for wells with rifampicin for CYP3A4 analysis and verapamil, *n* = 5 for the rest). Hepatocytes in spheroids and monolayers were exposed to these compounds for 72 h.

### 4.2. CYP450 and Viability Assays

CYP450 activity and viability were measured using separate luminescent assays (P450-Glo and CellTiter-Glo, Promega). After 72 h of drug exposure, media was removed and the spheroid was washed with PBS, with the plate first placed atop the magnetic drive to attract and hold spheroids on the well bottom while PBS was removed and added. Once washed, luciferin pro-substrates (Promega) were added to the wells, either: 3 μM Luciferin-IPA in serum-free media for wells with rifampicin (3A4) or verapamil; Luciferin-2B6 in PBS for wells with rifampicin (2B6) or ticlopidine; and Luciferin-1A2 in PBS for wells with omeprazole or α-napthoflavone. After incubation for 2 h, half of the media was aliquoted into separate white-walled microplates (Greiner Bio-One) and a luciferase reagent was added at an equal volume to the wells. In the original culture plate, a viability assay reagent (CellTiter-Glo, Promega) was added in equal volumes and incubated for 20 min. All plates were read for luminescence in a plate reader (Synergy 4, Biotek Instruments, Winooski, VT, USA). CYP450 activity was normalized to a luciferin standard.

### 4.3. Immunohistochemistry

After 72 h of compound exposure, spheroids were fixed in 4% paraformaldehyde (Electron Microscopy Sciences, Hatfield, PA, USA) for at least overnight, and stored at 4 °C in PBS. To embed spheroids in paraffin, the spheroids were first embedded in agarose by pouring a warm 2% solution directly into the 384-well plate, and upon cooling, removing the gel with the spheroid encapsulated within. This gel was further embedded in agarose to cover any exposed section of the spheroid. Finally, these gels were dehydrated, embedded in paraffin, and sectioned into 5 µm slices on glass slides according to standard protocols.

Prior to staining, sections were deparaffinized in xylene and rehydrated. Samples were blocked with 10% donkey serum buffer (Sigma-Aldrich) for 1 h, then stained with primary antibodies against either albumin (rabbit, Abcam, Cambridge, UK) or MRP2 (rabbit, Abcam) with an overnight incubation at 4 °C. Donkey serum was left on negative controls. The next day, the sections were washed in PBS, then fluorescently stained with a secondary antibody (AlexaFluor 488, ThermoFisher, Waltham, MA, USA). After washing with PBS, the nuclei were then counterstained by incubating the sections with DAPI (KPL, Gaithersburg, MD, USA) for 30 min. Once stained, the sections were washed again and mounted (Fluoromount-G, Southern Biotech, Birmingham, AL, USA). Images were taken of the stains on a fluorescent microscope (Axio Observer Z1, Zeiss, Jena, Germany). CYP450 activity was normalized to a luciferin standard.

### 4.4. Statistical Analysis

Statistical analysis was performed with one- and two-way analysis of variance (ANOVA) tests using statistical software (OriginPro 8.5, OriginLab, Northampton, MA, USA). Significance was defined as *p* < 0.05.

## 5. Conclusions

The study is a proof-of-concept of the use of magnetic 3D cell culture to assemble and culture hepatocyte spheroids and to analyze their CYP induction/inhibition in this model. This assay can also be expanded to more complex liver models and exploration of other DILI endpoints. This study lays the foundation for future applications of magnetic 3D cell culture for high-throughput DILI screening in spheroids, potentially improving accuracy in preclinical screening, while reducing the costs and adverse toxic events of progressing hepatotoxic compounds.

## Supplementary Materials

Supplementary materials can be found at www.mdpi.com/1422-0067/18/5/1085/s1.
