# Supplementary material for: Assembly of Hepatocyte Spheroids Using Magnetic 3D Cell Culture for CYP450 Inhibition/Induction
Source: Int J Mol Sci. 2017 May 18;18(5):1085. doi: 10.3390/ijms18051085 (PMC5454994; doi:10.3390/ijms18051085)
Supplement: Supplementary file 1 [file ijms-18-01085-s001.pdf]

Supplementary Materials: Assembly of Hepatocyte Spheroids Using Magnetic 3D Cell Culture for CYP450 Inhibition/Induction

Pujan K. Desai, Hubert Tseng and Glauco R. Souza

Table S1. *p*-Values for the effects of drugs and environment (2D v. 3D) on CYP activity.

	2D	3D
CYP3A4		
Rifampicin	2.1×10^{-6}	5.6×10^{-5}
2D v. 3D	1.7×10^{-4}	
Verapamil	0.048	6.7×10^{-12}
2D v. 3D	1.1×10^{-16}	
CYP2B6		
Rifampicin	3.2×10^{-15}	2.2×10^{-16}
2D v. 3D	0	
Ticlopidine	0.26	0.09
2D v. 3D	0	
CYP1A2		
Omeprazole	1.1×10^{-11}	4.7×10^{-5}
2D v. 3D	1.1×10^{-5}	
α-Napthoflavone	0.0022	9.6×10^{-7}
2D v. 3D	0	

Table S2. *p*-values for the effects of drugs and environment (2D v. 3D) on viability.

	2D	3D
Rifampicin	0.53	0.30
2D v. 3D	0.15	
Verapamil	0.002	0
2D v. 3D	0.03	
Rifampicin	3.8×10^{-5}	4.4×10^{-15}
2D v. 3D	0.22	
Ticlopidine	0.86	4.5×10^{-5}
2D v. 3D	0.45	
Omeprazole	1.4×10^{-11}	9.3×10^{-12}
2D v. 3D	0.054	
α-Napthoflavone	0.13	0.12
2D v. 3D	0.46	

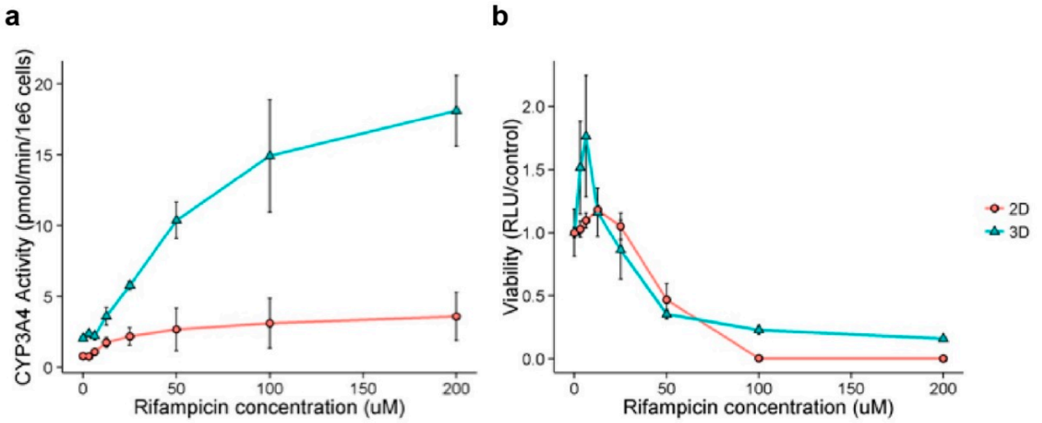

Figure S1. CYP3A4 activity and viability of iPS-hepatocytes after 72 h exposure to rifampicin. Human induced pluripotent stem cell-derived hepatocytes (iPS-hepatocytes, ReproCELL, Yokohama, Japan) were thawed and immediately either plated in 2D monolayers (75,000 cells/well, 96-well format) or printed into 3D spheroids (10,000 cells/spheroid, 384-well format) after 2 h incubation with the magnetic nanoparticles. iPS-hepatocytes were cultured for 24 h in thawing media and six days in maintenance media before replacing the maintenance media with a serum-free induction media containing rifampicin (0–200 μM) and a vehicle control (1% DMSO). After 72 h exposure to rifampicin and nine days after thaw, CYP3A4 activity and viability (ATP) were measured using luminescent assays on a plate reader. Spheroids show greater baseline and induced CYP3A4 activity than that of 2D monolayer culture ($n = 3$, $p < 0.001$ at 200 μM). Both cultures show cytotoxicity to high concentrations of rifampicin ($n = 3$, $p < 0.001$). Error bars represent standard error.
